# Etiology and management of cholangitis in pediatric liver transplant recipients: a systematic review

**DOI:** 10.1097/MOT.0000000000001285

**Published:** 2026-04-14

**Authors:** Alizée Hof, Robin Rey, Nathalie Rock, Arnaud G. L’Huillier

**Affiliations:** a Department of Human Medicine; b Platform of Clinical Research in Pediatrics, Gynecology and Obstetrics, Department of Paediatrics, Gynaecology and Obstetrics, Faculty of Medicine, University of Geneva; c Swiss Paediatric Liver Center, Department of Woman, Child and Adolescent Health, Geneva University Hospitals and University of Geneva; d Pediatric Infectious Diseases Unit, Department of Woman, Child and Adolescent Health, Geneva University Hospitals; e Department of Pediatrics, Gynecology and Obstetrics, Faculty of Medicine, University of Geneva, Geneva, Switzerland

**Keywords:** cholangitis, liver transplant, pediatric, treatment

## Abstract

**Purpose of review:**

Children undergoing liver transplantation are highly vulnerable to infections. Cholangitis is a potential post-transplant complication requiring broad-spectrum anti-infective therapy, raising concerns about antimicrobial resistance in this immunosuppressed population. We conducted a systematic review to evaluate antimicrobial management of post-transplant cholangitis in pediatric patients.

**Recent findings:**

Nine heterogeneous studies were included. Definitions of cholangitis varied widely, combining clinical, laboratory, imaging, and microbiological criteria, highlighting the need for standardization. Gram-negative bacteria predominated, particularly *Klebsiella* spp., *Pseudomonas aeruginosa*, and *Escherichia coli*. Prophylaxis commonly relied on broad-spectrum antibiotics, and initial treatment was empirical in all studies, with occasional adjustment based on microbiological results. First-line therapies included piperacillin–tazobactam and third-generation cephalosporins. Second-line regimens involved agents from various antibiotic classes, including glycopeptides, lipopeptides, aminoglycosides, and fluoroquinolones, as well as antifungals. Meropenem was used as either first-line or second-line therapy.

**Summary:**

Improved characterization and standardized reporting of pathogens and treatments are needed to guide targeted antimicrobial strategies and limit multidrug-resistant organisms. More detailed and harmonized data reporting is essential to optimize management of post-transplant cholangitis in children.

## INTRODUCTION

Liver transplantation is a life-saving therapeutic option for children with end-stage liver disease. Biliary atresia is the most common indication for pediatric liver transplantation (PLT; approximately 50%), followed by metabolic disorders (15%), fulminant hepatitis, cholestatic disorders (e.g. Alagille syndrome, progressive familial intrahepatic cholestasis), and malignancies [1,2]. Pediatric liver transplant recipients are vulnerable to infections due to multiple factors such as immunosuppression, long hospitalization, indwelling catheters, and procedures such as surgery and post-transplant cholangiography for bile drainage [3,4]. Despite significant advances in surgical technique, perioperative care and antimicrobial prophylaxis, infectious complications remain a major source of morbidity and mortality in pediatric liver transplant recipients, accounting for half of death in the immediate postoperative period [3,5]. Among infectious complications, the incidence of post-transplant cholangitis ranges from 1 to 10%, depending on the type of biliary reconstruction [6]. Cholangitis results from biliary obstruction or stasis, which facilitate the ascent of enteric pathogens into the biliary tree. Clinical manifestations are ranging from mild cholangitis to severe sepsis or septic shock, and they may also compromise graft function [7].

Management of post-transplant cholangitis involves the use of broad-spectrum anti-infective agents to cover the most common pathogens such as *Escherichia coli*, *Klebsiella* spp., *Enterococcus* spp., and *Pseudomonas aeruginosa* [4,8,9]. However, this empiric approach has raised concerns regarding antimicrobial resistance, especially in immunosuppressed hosts who are particularly at risk for multidrug-resistant organisms because of their increased healthcare exposure, their limited ability to clear infections, and their more frequent exposure to antibiotics. Therefore, optimizing anti-infective strategies requires a balance between adequate empiric coverage and antimicrobial stewardship.

To our knowledge, no systematic review nor guidelines exist on the antimicrobial management of post-transplant cholangitis in children. The present systematic review aims to examine the antimicrobial management of acute cholangitis following pediatric liver transplantation by identifying the most common etiologic agents and describing empiric and targeted anti-infective strategies used in this context. 

**Box 1:**
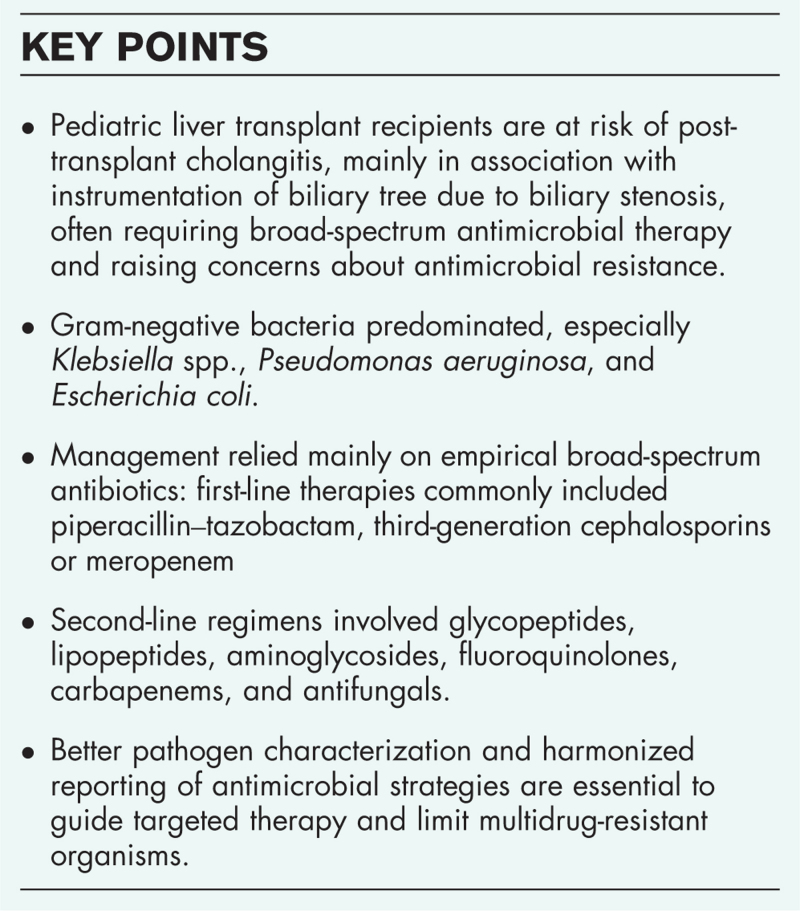
no caption available

## MATERIALS AND METHODS

This systematic review was conducted following the Preferred Reporting Items for Systematic Reviews and Meta-Analyses (PRISMA) guidelines (checklist is available in Supplementary Material 3.

### Literature search

Literature search was done using PubMed, Embase, and Web of Science databases in December 2025, using the keywords ‘Pediatric’, ‘Liver transplant’, ‘Cholangitis’, ‘Anti-infective treatment,’ and their relevant synonyms combined with Boolean operators, adapted to the syntax and indexing of each database. The complete search strategy is available in Supplementary Material 1. Studies describing the anti-infective treatment of acute cholangitis in liver transplant recipients aged less than 18 years were included. Items were excluded if the article was not available, or in case of informal publication (letter, commentary, editorial, abstract only). No restrictions in terms of language, year of publication, or study design were applied. First, one reviewer (A.H.) screened all articles by title and abstract. Then, two reviewers independently (A.H. and R.R.) screened the remaining articles’ full texts and reached consensus on the final set of included studies. Two other reviewers (N.R. and A.L.) resolved uncertainties regarding article selection at the final stage of the process.

### Data

Data were extracted using a predefined form that captured article characteristics (authors, year of publication), study design and population (age, indication for liver transplant, biliopathy, definition of cholangitis), post-transplant antibioprophylaxis, pathogens and source of infection described, anti-infective agents used, and time between liver transplant and cholangitis. When available, details on the route of administration, duration, and dosage of treatment were also retrieved. Some authors were contacted for additional data, information or details regarding their publications.

### Risk of bias and synthesis

The risk of bias was assessed by two reviewers (A.H. and R.R.) using the Newcastle–Ottawa Scale (NOS) for cohort studies and the Joanna Briggs Institute (JBI) Critical Appraisal tool for case report and case series. No meta-analysis was done and findings for all the outcomes of interest are presented in descriptive tables. The risk of bias is presented in tables for each individual study.

## RESULTS

### Study selection

**FIGURE 1:**
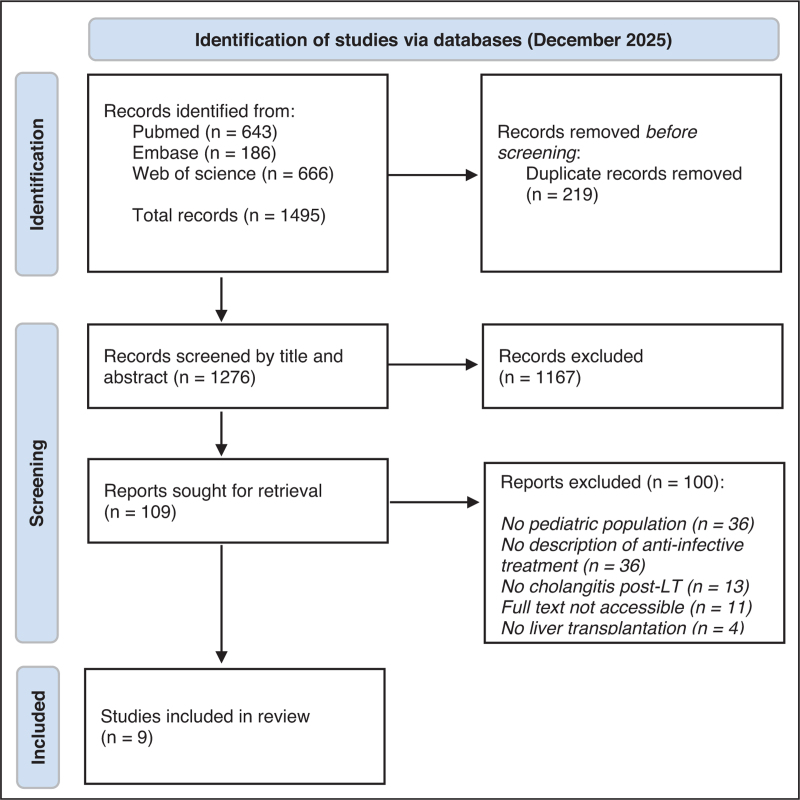
PRISMA flow diagram. LT, liver transplantation.

A total of 1495 publications were identified across the three databases. After removing 219 duplicates, 1167 were excluded during title and abstract screening, and 100 after full text review, resulting in nine studies included in the final selection (Fig. 1).

### Study characteristics

The characteristics of the included studies are summarized in Table 1 and in Supplementary Material 2. All included studies were published between 2016 and 2025. Of the nine studies, seven were retrospective [10–16], one was a case series [17], and one was a case report [18]. Total cohort sizes ranged from 27 to 210 patients, with the number of PLT recipients varying widely (from 2 to 188 patients). The number of cholangitis episodes reported per study ranged from 7 to 43. Median or mean age of children reported in these studies mostly ranged between 2 and 5 years. Among studies reporting indication for liver transplantation, biliary atresia was the most common diagnosis in four of seven studies [10–13]. Other frequent indications included genetic disorders, hepatitis, and oncologic disorders. Two studies did not report indication for liver transplantation [15,17].

**Table 1:** Characteristics of the included studies

Number	Author; design; year	Population; age	Indication for liver transplant	Definition of cholangitis	Time since LT; type of culture	Pathogens (by frequence)	Antibioprophylaxis	Anti-infective agents
1	Ashkenazi-Hoffnung *et al.* Retrospective study; 2016	PLT: 44 hospitalization for fever: 133 episodes post-transplant Cholangitis among the hospitalizations: 28	Whole cohort: Biliary atresia: 27 Hepatitis: 6 Genetic disorder: 9 Oncologic disorder: 0 Other: 2	(A) signs of systemic inflammation (B) signs of cholestasis (C) imaging (biliary tract dilatation and/or evidence of etiology on imaging) Suspected: (A) and [(B) or (C)] Confirmed: (A) and (B) and (C)	Whole cohort: <1 month: 3 1 month–1 year: 39 1–5 years: 58 > 5 years: 33	Cholangitis: *E. coli*: 3 including *MDR*: 2 *Enterobacter* spp*.:* 1 *Enterococcus* spp*.:* 1 *E. coli* and *Klesbiella* spp.: 1 *Klesbiella* and *Enterococcus* spp*.:* 1 *Pseudomonas* and *Enteroccocus* spp*.:* 1 Unknown: 20	Prophylaxis post LT: piperacillin/tazobactam and gentamicin	Empirical antibiotic treatment for all cholangitis*
		Whole cohort: Median (SD): 4.7 (5.07) years at admission			Blood			
2	Lin *et al.* Retrospective study; 2016	PLT: 188 Post-transplant cholangitis (nonsuppurative): 9	Among PLT cholangitis: Biliary atresia: 6 Other: 3	Histology (non-suppurative cholangitis): increased bile ductule profiles at the edges of portal tracts, bile plugs within ductules, paucity of acute inflammation in portal tracts, and normal numbers of interlobular bile duct profiles with nonreactive morphology*	Whole cohort: Mean (SD): 0.9 months (0.59 months) between LT and biopsy	Whole cohort: *P. aeruginosa* *K. pneumoniae* *Enterococcus* spp. *Bacteroides* spp *Citrobacter* spp. *S. aureus S. pneumoniae* *C. albicans* (NI about frequency)	NI	Antibiotic treatment*
		Cholangitis cases: Mean (SD): 5.6 (5.4) years			Blood Body fluids Wounds Pus Lines			
3	Sansotta *et al.* Retrospective study; 2021	PLT: 50 PTC post-PLT: 157 Post-transplant cholangitis after PTC: 70	Whole cohort: Biliary atresia: 33 Genetic disorders: 7 Other: 10	(A) Fever (>38.5°C) (B) Elevated inflammatory markers after PTC Defined as: (A) and (B)	NI	Whole cohort: *E. faecium:* 15 including *VRE:* 3 *P. aeruginosa:* 10 *E. coli:* 5 including *ESBL:* 2 and *KPC:* 1 *K. pneumoniae:* 4 including *ESBL:* 2 *C. albicans:* 4 *S. aureus:* 2 *E. aerogenes:* 2 including *ESBL:* 2 *E. faecalis:* 2 *E. cloacae:* 1 including *ESBL:* 1 *A. baumanii:* 1	1st line in PTC (*n* = 135): cefotaxime: 73 piperacillin-tazobactam: 46 ciprofloxacin: 16 2nd line in PTC (*n* = 22*): meropenem daptomycin vancomycin	Antibiotherapy continued after 115/157 procedures (70/115 for cholangitis) 1st line: 81/115* 2nd-line (34/115): vancomycin: 13 meropenem: 10 daptomycin and/or linezolid: 6 aminoglycosides: 4 fluconazole and micafungin: 2
		Whole cohort: Median (IQR): 3.05 (3–9) years at PTC			Blood performed 70/70 of cholangitis, positive in 15/70 Bile: 30/70 of cholangitis, positive in 20/30			
4	Soria-Navarroa *et al*. Retrospective study; 2022	Children: 37 including 26 PLT NSBTM: 113, including 77 after PLT Cholangitis (pre- and post-transplant): 27 Cholangitis (among PLT): 7 episodes (in 5 patients) **	Among PLT: Biliary atresia: 11 Oncologic disorder: 5 Others: 10	(A) systemic inflammation (B) cholestasis (C) imaging abnormalities (biliary dilatation or evidence of its cause) Defined as: (A) and (B) and (C)	PLT subgroup: Median (IQR): 8 months (2 months to 2 years) between LT and procedure	NSBMT-related infections: *Enteroccocus* spp.: 12 *Klesbiella* spp.: 9 *E. cloacae:* 4 *P. aeruginosa*: 4 *S. maltophilia:* 4 *Candida* spp.: 3 *E. coli:* 2 *C. freundii:* 1 Cholangitis among PLT: ** P. aeruginosa: 1 *E. coli* and *E. faecium: 1* *ESBL-producing K. pneumoniae: 2* *AmpC-producing K. oxytoca: 1* *E. faecalis: 1*	Periprocedural prophylaxis (no infection at time of procedure, *n* = 69): piperacillin-tazobactam: 53 meropenem: 9 cefoxitin: 2 ciprofloxacin: 1	Antibiotic treatment for infection during NSBTM (*n* = 44): piperacillin–tazobactam 16 meropenem 13 combined antibiotherapy* 12 Cholangitis among PLT: ** Empirical treatment: piperacillin-tazobactam Targeted treatment: meropenem or ertapenem (for *ESBL*), teicoplanin (for *E. faecium*), piperacillin-tazobactam in case of negative cultures
		Whole cohort: Median (IQR): 4 (1–8) years at time of NSBTM			Blood in 29/32 NSBMT-related infections, positive in 9/29 (polymicrobial in 2/9) Bile in 18/32 NSBMT-related infections, positive in 16/18 (polymicrobial in 9/16) Blood in 7/7 PLT cholangitis, positive in 3/7** Bile in 5/7 PLT cholangitis, positive in 5/5**			
5	Ying *et al*. Retrospective study; 2020	LT: 210 Post-transplant infections: 196 Post-transplant cholangitis: 43/196	Whole cohort: Viral hepatitis: 139 Inherited liver disease: 24 Toxic related liver disease: 13 Autoimmune liver diseases: 11 Hepatocellular carcinoma: 11 Cirrhosis: 3 Others: 9	(A) fever, right upper quadrant pain, and elevation of liver chemistry (B) evidence of cholangitis on liver biopsy (C) isolation of the same organism from drain and blood Defined as: (A) and [(B) or (C)]	Whole cohort: Time to infection (all infections combined): 0–1 month post LT: 153/196 1–3 months: 26/196 > 3 months: 17/196	Cholangitis: *S. maltophilia:* 14% *K. pneumoniae:* 12% *P. aeruginosa:* 9% *E. faecium:* 9% *Staphylococcus* spp.: 7% Funghi*: 9% *A. baumanii:* 2%	Prophylaxis post LT: based on donor situation and culture*	Empirical treatment, then tailored based on susceptibility testing*
		Whole cohort: Median (range): 50 (0.6–69) years at transplant			Blood culture Bile culture			
6	Zhang *et al*. Retrospective study; 2018	Children: 27 including 2 PLT Cholangitis: 27 (surgery-related: 19; surgery-unrelated: 8)	NI	(A) fever (B) increased WBC/CRP (C) increased transaminases (D) bile sludge or gallstones observed by abdominal ultra sonography Suspected (after biliary atresia surgery): (A) Confirmed (after biliary atresia surgery or PLT): (A) and [(B) and/or (C)] Confirmed (no surgery): (A) and [(B) and/or (C)] and (D)	NI	Whole cohort: *E. coli:* 2 *K. pneumoniae:* 1	NI	1st line: 3rd generation cephalosporin 2nd line: meropenem
		Whole cohort: Range: 3 months–13 years *			Blood			
7	Işik *et al*. Retrospective study; 2024	PLT: 155 Neutropenia: 60 Cholangitis: 7 among the 60 neutropenia	Neutropenic group: Genetic disorder: 26 Biliary atresia: 16 Hepatitis: 6 Oncologic disorder: 2 Other: 10	NI	NI	NI	NI	Neutropenia group: IV antibiotics: 60% oral antibiotics: 5%
		Neutropenia group: Mean (SD): 4.82 (4.93) years			Blood			
8	Garcia-Boyano *et al*. Case series; 2024	Children treated with CZA: 18 PLT: 8 Cholangitis: unclear	NI	NI	NI	Whole cohort: *K. pneumoniae:* 16 including *OXA48:* 15 *P. aeruginosa:* 1	NI	Initial treatment (whole cohort): Meropenem and amikacin: 6 Meropenem: 2 Piperacillin–tazobactam: 2 Meropenem and ceftazidime: 1 Meropenem and levofloxacin: 1 All patients eventually treated with CZA
		Whole cohort: Median (IQR): 21 (10–43) months Range: 87 days - 15.4 years) at treatment onset			Blood Bile Ascitic fluid			
9	Odenthal *et al*. Case report; 2025	1	Hepatoblastoma	Clinical status: jaundice, pruritus, cholic stools Laboratory: transaminases, gamma-GT, bilirubin Ultrasound: intra and extrahepatic biliary ducts dilatation	7 months between LT and initial PTC; follow-up PTC performed 6 months after initial one	NI	NI	Meropenem
		5 years						

(*) Not otherwise specified; (**) additional information provided by the authors upon request; *A., Acinetobacter; C., Citrobacter*; CZA, ceftazidime–avibactam; *E. coli*, *Escherichia coli*; *E., Enterococcus*; ESBL, extended spectrum beta-lactamase producer; IQR, interquartile range; IV, intravenous; *K.*, *Klebsiella*; KPC, *Klebsiella pneumoniae* carbapenemase; LT, liver transplantation; MDR, multi-drug resistant; NI, no information; NSBTM, nonsurgical manipulation of the biliary tract; OXA-48, Oxacillinase-48; *P.*, *Pseudomonas*; PLT, pediatric liver transplantation; PTC, percutaneous transhepatic cholangiography; *S.*, *Staphylococcus*; SD, standard deviation; VRE, vancomycin-resistant *Enterococcus*.

### Definition of cholangitis definition and time between transplantation and infection

The definition of cholangitis varied across studies but generally required a combination of clinical, laboratory, imaging, and/or microbiological criteria. Most articles (4/9 studies) relied on three parameters [10,13,15,18]: systemic inflammation (defined by fever, chills, elevated white blood cell count or elevated C-reactive protein); cholestasis (defined by jaundice or abnormality of the liver function tests); and biliary abnormalities on imaging (intrahepatic and/or extrahepatic bile duct dilatation). One study defined cholangitis based on histological criteria (non-suppurative cholangitis) [11], another based on fever and elevated inflammatory markers [12], and one study required microbiological confirmation of pathogens in addition to clinical criteria [14]. Two articles did not provide a definition of cholangitis [16,17]. The time between liver transplantation and cholangitis was highly heterogeneous but a vast majority of infections occurred in the first year following liver transplantation or after a liver-related procedure (e.g. percutaneous transhepatic cholangiography [PTC] or endoscopic transpapillary procedures [ETP]).

### Pathogens

Pathogen identification relied primarily on blood cultures in eight of nine studies [10–17], obtained in cases of suspected cholangitis. Four studies additionally used bile culture for pathogen identification [12–14,17], while two studies also included cultures from ascitic fluid as well as from wounds, pus, and intravascular lines [11,17], not specific to cholangitis but to infections overall. Pathogens were not consistently documented and when documented, discrimination between cholangitis and other infections were not necessarily provided at the pathogen level, only two studies [10,13] reported specifically germs identified in PLT cholangitis. In these two studies [10,13], Gram-negative bacteria were most frequently identified, especially *Klebsiella* spp., *P. aeruginosa, E. coli*, and *Enterobacter* spp., while Gram-positive bacteria were less frequent and mainly included *Enterococcus* spp. such as *Enterococcus faecium* and *Enterococcus faecalis.*

### Antimicrobial prophylaxis and treatment

Three studies reported antibiotic prophylaxis: two addressed periprocedural prophylaxis (PTC and ETP) [12,13], and one described prophylaxis following liver transplantation [10]. Prophylactic regimens mainly involved broad-spectrum antibiotics, including piperacillin–tazobactam, third-generation cephalosporins, carbapenems, and ceftazidime–avibactam. Initial antibiotic management of cholangitis was empirical in all studies. Five studies reported adaptation of therapy according to microbiological results [12–15,17]. First-line treatment included piperacillin–tazobactam [13], third-generation cephalosporins [15], meropenem [18], or any of these three agents [17]. Second-line therapies comprised vancomycin, daptomycin, amikacin, linezolid, ceftazidime or levofloxacin, meropenem, and antifungal agents. None of the included studies reported the rate of cholangitis resolution or mortality attributable to post-transplant cholangitis, precluding any analysis of these outcomes.

### Risk of bias

The overall risk-of-bias assessment is presented in Table 2. The main limitation identified in the majority of studies (six of seven studies) [10–16] concerned the comparability domain, as study designs did not allow comparison with non-exposed or healthy cohorts, nor did they adequately adjust for confounders when analyzing risk factors for infection or cholangitis after liver transplantation. In addition, Table 3 summarizes the principal limitations of the included studies in relation to our research question, particularly the limited reporting of pathogens and anti-infective treatments specific to cholangitis in PLT recipients.

**Table 2:** Risk-of-bias assessment of the included studies, with the Newcastle–Ottawa Scale for retrospective studies and Joanna Briggs Institute Critical Appraisal tool for case report and case series

Newcastle–Ottawa Scale	Selection (0–4)	Comparability (0–2)	Exposure (0–3)	Total (0–9)
1/Ashkenazi-Hoffnung *et al*.	☆☆		☆☆☆	5/9
2/Lin *et al*.	☆☆☆		☆☆☆	6/9
3/Sansotta *et al*.	☆☆		☆☆☆	5/9
4/Soria-Navarroa *et al*.	☆☆☆	☆☆	☆☆☆	8/9
5/Ying *et al*.	☆☆		☆☆☆	5/9
6/Zhang *et al*.	☆☆		☆☆☆	5/9
7/Işik *et al*.	☆☆☆		☆☆☆	6/9

Joanna Briggs Institute Critical Appraisal tool	Responses to questions		
	Yes	Unclear	No	Overall
8/Garcia-Boyano *et al*.	9/10	1/10	0/10	Good
9/Odenthal *et al.*	6/8	2/8	0/8	Good

**Table 3:** Limits of the included studies for the research objectives of this systematic review

	Pathogen		Treatment		
Number/author	Cholangitis vs. other	LT vs. no LT	Cholangitis vs. other	LT vs. no LT	Others
1/Ashkenazi-Hoffnung *et al*.	S	S	S	S	Antibiotic treatment not specified
2/Lin *et al*.	U	S	S	S	Antibiotic treatment not specified Pathogen frequency not reported Indication for PLT unclear
3/Sansotta *et al*.	U	S	U	S	First-line antibiotic treatment not specified
4/Soria-Navarroa *et al*.	S**	S**	S**	S**	
5/Ying *et al*.	U	S	U	S	No discrimination regarding pathogens or treatment between pediatric vs. adult LT recipients Antibiotic treatment not specified
6/Zhang *et al*.	U	U	U	U	Indication for PLT unclear Age of the population unclear Few pathogens reported
7/Işik *et al*.	–	–	U	S	Antibiotic treatment not specified Pathogen frequency not reported Cholangitis definition unclear Selection bias (neutropenic infections only)
8/Garcia-Boyano *et al*.	U	U	U	U	Indication for PLT unclear Cholangitis definition unclear Selection bias (patients treated with CZA)
9/Odenthal *et al*.	–	–	S	S	No pathogen identified

(**) Additional information provided by the authors upon request. CZA, ceftazidim–avibactam; S, specified; U, unspecified.

## DISCUSSION

To our knowledge, this is the first systematic review focusing on the etiology and management of cholangitis following PLT. Even though almost 1500 articles were screened, very few articles could be included in the review, confirming the paucity of data on the topic and the heterogeneity of the included studies in terms of population, cholangitis definitions, pathogens, and treatment.

Unlike the recently proposed definition of cholangitis following Kasai hepatoportoenterostomy in biliary atresia, which included clinical and laboratory/imaging criteria [19,20], there is no standardized definition of post-transplant cholangitis in either the pediatric or adult population. Most included articles in our review defined cholangitis as a triad of systemic inflammation, cholestasis, and biliary abnormalities on imaging [10,13,15,18]. A standardized definition of cholangitis following PLT would allow consistent identification, reporting, and management of this relatively rare condition across studies and settings.

The age of reported pediatric cohorts varied between 2 and 5 years, suggesting that cholangitis occurs more frequently in infants and in the first years following liver transplantation, which is probably multifactorial. First, post-transplant infections are more frequent in infants than older children [21], likely because of the immaturity of their immune system, the absence of pathogen-specific immunity at time of transplant. Second, the first year(s) following PLT are at highest risk of infection because of a high net state of immunosuppression, immune dysregulation [22]. Third, the frequency of cholangitis in infants is at least partially related to the fact that the most common indication for PLT is biliary atresia, a condition where children are usually transplanted around 1 year old [23]. In young children, direct choledocho-choledochal anastomosis is often not feasible, requiring a Roux-en-Y hepaticojejunostomy, which is associated with a higher risk of biliary anastomotic strictures and the need for subsequent interventions, and procedure at risk of infections. Unfortunately, the impact of procedures such as post-transplant cholangiography is not clearly defined across the studies included in this review even if PTC in PLT seem to have a high rate of infectious complication, including cholangitis [12,24].

Gram-negative bacteria were the most frequent pathogens identified, in particular *E. coli, Klebsiella* spp. and *P. aeruginosa.* This is in line with previous data showing that in adult liver transplant recipients with infectious complications including cholangitis, *E. coli, P. aeruginosa, K. pneumoniae, and E. faecium* are the most frequent germs identified [25–27].

Culture of biological specimens play a crucial role in pathogen identification by allowing a step-down to a more targeted anti-infective therapy. In our systematic review, blood cultures were performed systematically in eight of the included studies, reflecting the relatively non-invasive character of this procedure. However the sensitivity of blood culture in our systematic review ranged between 21 and 43% [12,13] for the diagnosis of cholangitis when reported. In contrast, bile culture had a substantially higher sensitivity in our review, ranging between 67 and 100% when reported [12,13]. They were, however, performed in less than half of the studies, reflecting the challenge to obtain bile culture in the absence of indwelling drains. The difference in sensitivity between blood and bile culture for the diagnosis of cholangitis is in line with data showing a positive rate of blood and bile cultures of 35% and 85%, respectively in the general population affected by cholangitis [28]. However, bile culture results should be interpreted with caution as the colonization of indwelling bile drains can be associated with false-positive results.

Antibiotic treatment for cholangitis was initiated empirically across studies and adapted according to microbiological identification whenever available. The frequent use of broad-spectrum antibiotics both for prophylaxis and for treatment of cholangitis likely reflect a combination of limited pathogen-specific data, and concerns for severe complications in these vulnerable patients. Interestingly, duration of antimicrobial treatment for cholangitis was rarely reported. The absence of evidence regarding optimal antimicrobial regimen and duration confirms the need for a standardized definition to allow for comparability between centers and consequently generate robust data through dedicated studies, which could enable more tailored antimicrobial strategies, maintaining clinical efficacy while potentially limiting the emergence of drug resistance.

This study had several limitations. First, the number of included studies was limited. Second, pathogen frequency was commonly not reported. Third, discrimination of pathogens and/or antimicrobial treatment was commonly not performed between cholangitis and other infections, as well as between transplanted and non-transplanted (pediatric) patients. Then, anti-infective agents were insufficiently described, especially regarding length, posology, and route of administration. Finally, several studies were restricted to specific subgroups like neutropenic patients or patients treated with specific molecules.

## CONCLUSION

This systematic review underscores the limited and heterogeneous evidence on etiology and antimicrobial management of cholangitis following PLT. Standardized definitions are urgently needed generate more evidence regarding antimicrobial regimen and duration in this vulnerable population.

## Acknowledgements

*None*.

### Financial support and sponsorship

*None.*

### Conflicts of interest

*There are no conflicts of interest.*

## Supplementary Material

## REFERENCES AND RECOMMENDED READING

Papers of particular interest, published within the annual period of review, have been highlighted as:

▪ of special interest
▪▪ of outstanding interest
